# Human Nmnat1 Promotes Autophagic Clearance of Amyloid Plaques in a *Drosophila* Model of Alzheimer’s Disease

**DOI:** 10.3389/fnagi.2022.852972

**Published:** 2022-03-24

**Authors:** Yi Zhu, Amanda G. Lobato, R. Grace Zhai, Milena Pinto

**Affiliations:** ^1^Department of Molecular and Cellular Pharmacology, University of Miami Miller School of Medicine, Miami, FL, United States; ^2^Department of Neurology, University of Miami Miller School of Medicine, Miami, FL, United States

**Keywords:** APP, NAD, *Drosophila*, autophagy, aggregates

## Abstract

Alzheimer’s disease (AD) is a progressive neurodegenerative disease characterized by irreversible cognitive decline with limited therapeutic approaches. We characterized a *Drosophila* model of amyloid pathology that expresses human amyloid-beta precursor protein (APP^695^) and β-site APP cleaving enzyme (BACE) in the nervous system. Our model recapitulates *in vivo* the age-dependent accumulation of BACE-derived C-terminal fragment (CTF) and amyloid plaques in the brain, one of the key pathological hallmarks of AD. Using this model, we assessed the effects on plaque formation of Nicotinamide mononucleotide adenylyltransferase (Nmnat), an evolutionarily conserved nicotinamide adenine dinucleotide (NAD^+^) synthase involved in cellular metabolism and neuroprotection. We compared the effects of overexpression of *Drosophila* Nmnat (dNmnat), human Nmnat1 (hNmnat1), human Nmnat2 (hNmnat2), and human Nmnat3 (hNmnat3), and observed that hNmnat1 has the highest efficacy in reducing amyloid aggregation and APP-CTF accumulation. Interestingly, we demonstrated that overexpression of hNmnat1 reduces amyloid plaques by promoting autophagic clearance. Our findings uncover a role of hNmnat1 in amyloid clearance and suggest an exciting neuroprotective potential of hNmnat1 in amyloid pathology.

## Introduction

Alzheimer’s disease (AD) is a neurodegenerative disorder that leads to progressive memory loss and cognitive decline and represents the most common form of late-onset dementia (LaFerla et al., 2007; Masters et al., 2015). Two of the main pathological features of AD are amyloid plaques, extracellular insoluble aggregates composed of amyloid-beta fragments (Aβ), and neurofibrillary tangles, intracellular accumulation of hyperphosphorylated Tau (Ingelsson et al., 2004; LaFerla et al., 2007). Together, amyloid pathology and Tau aggregates drive the neuronal impairment that widely affects the cerebral cortex and hippocampus of AD patients (Masters et al., 2015). The effects of these plaques and tangles on cellular functions include mitochondrial dysfunction, synaptic degeneration, and autophagy dysfunction (Ingelsson et al., 2004).

Autophagy is a cellular pathway that controls proteostasis by sequestering and delivering protein aggregates and cellular organelles to lysosomes for degradation. Multiple stages of autophagy are disrupted in AD. *Postmortem* brain samples of AD patients show decreased levels of Beclin 1, which is essential for autophagy initiation by recruiting membranes to form autophagosomes before fusion to lysosome. Heterozygous deletion of *Beclin 1* in transgenic mice expressing human amyloid precursor protein (hAPP) shows compromised neuronal autophagy and accelerated neurodegeneration due to Aβ accumulation (Pickford et al., 2008). Enhancing autophagy through overexpression of Atg8a, a homolog of mammalian LC3, in a *Drosophila* model of AD increases stress resistance and extends lifespan (Tsakiri et al., 2021). Targeting autophagy and improving protein quality control shows promising therapeutic potential in various neurodegenerative disorders including AD, Huntington’s disease (HD), and Parkinson’s disease (PD) (Nixon, 2013).

Activation of autophagy is involved in the neuroprotection conferred by overexpression of Nmnat (Nicotinamide mononucleotide adenylyltransferase), an evolutionarily conserved rate-limiting enzyme involved in the synthesis of NAD^+^. In a hypertensive glaucoma model, Nmnat protects against optic nerve degeneration through increasing autophagic flux in retinal ganglion cells (Kitaoka et al., 2013). Under hypoxic stress, Nmnat functions upstream of autophagy to mitigate the damage incurred by dendrites in neurons (Wen et al., 2013). We have previously shown that in a *Drosophila* model of HD, mutant Huntingtin (Htt) aggregation impairs autophagic pathway, while overexpressing Nmnat promotes autophagic clearance of Htt aggregates and protects against neurodegeneration (Zhu et al., 2019). *Drosophila* has a single *Nmnat* gene (*dNmnat*), and its loss-of-function causes post-development photoreceptor neurodegeneration (Zhai et al., 2006). Overexpression of dNmnat rescues neurodegeneration caused by aggregation of toxic proteins including Tau (Ali et al., 2012; Ma et al., 2020), Ataxin (Zhai et al., 2008; Ruan et al., 2015), and Htt (Zhu et al., 2019).

In mammals, three *Nmnat* genes produce three different Nmnat protein isoforms with distinct subcellular localizations: Nmnat1 in nuclear, Nmnat2 is present in the Golgi and in the cytoplasm, and Nmnat3 is mainly mitochondrial (Brazill et al., 2017). Nmnat1 is ubiquitously expressed and is one of the most studied isoforms of Nmnat. Its expression is protective against axon degeneration caused by mechanical or toxic insults. Mutations in this gene cause a recessive, early form of blindness genetically defined as Leber Congenital Amaurosis 9 (LCA9) (Falk et al., 2012). Nmnat1 knockout mice do not survive birth, while heterozygous mice develop normally without detectable neurodegeneration or axonal pathology (Conforti et al., 2011). On the other side, Nmnat1 overexpression reduces early behavioral impairment in a mouse model of tauopathy (Rossi et al., 2018) and reverses the loss of tyrosine hydroxylase (TH) neurons in the nigrostriatal pathway of the 3xTgAD mice (Jiang et al., 2021). Nmnat2 is predominantly neuronal and has the most influence over axon survival under physiological conditions: depletion of Nmnat2 causes a primary axonal phenotype (Gilley and Coleman, 2010) and mice lacking Nmnat2 have a severe axon outgrowth defect resulting in axon truncation in the peripheral and central nervous system that is incompatible with postnatal survival (Gilley et al., 2019). Reduced Nmnat2 mRNA levels are seen in AD, PD, and HD patients (Ali et al., 2016), and in a mouse model of tauopathy (Ljungberg et al., 2012). Nmnat3 is also ubiquitously expressed but predominantly present in the liver, heart, red blood cells, and skeletal muscle (Brazill et al., 2017). Nmnat3 is upregulated upon hypoxia-ischemia insult (Galindo et al., 2017). Overexpression of Nmnat3 prevents cortical and hippocampal tissue loss, while Nmnat3 knockdown causes neurodegeneration and increases excitotoxic cell death (Galindo et al., 2017).

In this report, we analyzed the effect of expressing dNmnat and human Nmnat1, 2, and 3 in suppressing the proteotoxic phenotype in a *Drosophila* model of Aβ pathology. We focused on the APP processing and Aβ plaque deposition in the brain and described the regulation of autophagy in Nmnat-facilitated clearance of amyloid aggregation.

## Results

### Neuronal Expression of APP and BACE1 Leads to an Age-Dependent Accumulation of Amyloid Aggregates in the Brain

**FIGURE 1:**
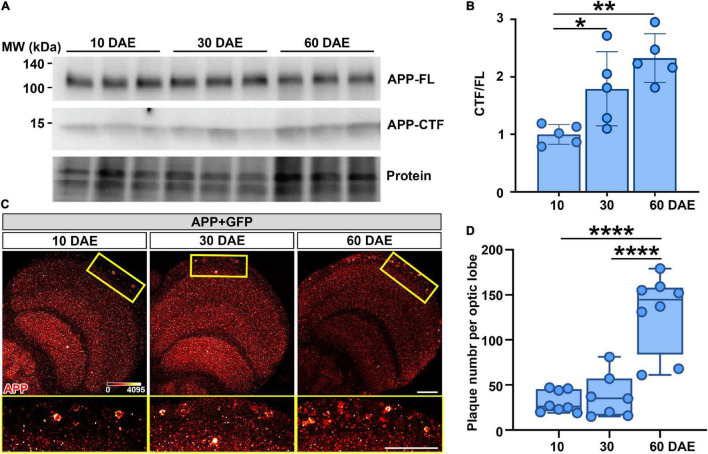
Neuronal expression of APP and BACE1 leads to an age-dependent amyloid aggregation in the brain. **(A)** Western blot analysis showing full-length amyloid precursor protein (APP-FL) and C-terminal fragment (APP-CTF) of 10, 30, and 60 DAE flies expressing APP. Stain-Free imaging of a representative portion of the membrane is presented to show the protein loading. **(B)** Quantification of CTF/FL ratio in each group. Data are expressed as mean ± SD. *n* = 5/group. One-way ANOVA with Bonferroni’s *post hoc* test. *P < 0.05, **P < 0.01. **(C)** Top row: fly brains at 10, 30, and 60 DAE stained with antibody anti APP (heatmap). Bottom row: higher magnification images of the boxed areas in the top row. Scale bars = 30 μm. **(D)** Scatter plot showing quantification of the number of amyloid plaques in panel **(C)**. The whiskers represent the minimum and maximum values of the dataset. One-way ANOVA with Bonferroni’s *post hoc* test. *n* = 7–8/group, ****P < 0.0001.

The age-dependent accumulation of Aβ plaques and the ensuing oxidative and inflammatory responses are some of the key pathogenic factors of Alzheimer’s disease (LaFerla et al., 2007; Masters et al., 2015). The plaque-forming amyloid protein is produced by an initial cleavage of the APP by β-secretase (β-APP-cleaving enzyme-1 or BACE1) to generate a membrane-bound C-terminal fragment (CTF99), and a subsequent cleavage by γ-secretase to generate amyloidogenic peptides Aβ_1–40_ and Aβ_1–42_ (LaFerla et al., 2007). To model amyloid pathology in *Drosophila*, we used a pan-neuronal constitutive driver *elav-GAL4* to express a Myc-tagged APP^695^ isoform of the human APP (*UAS-APP695-N-myc*), which is the most highly expressed isoform in neurons (Belyaev et al., 2010), together with BACE1 (*UAS-BACE1*) in the *Drosophila* nervous system. To determine if APP and BACE1 were expressed and if the secretase was functional, we performed a western blot analysis using an antibody that recognizes the C-terminal residues of APP (amino acid 676–695). The antibody recognizes the full-length (FL) APP (110 kDa) and the CTF (15 kDa) that remain membrane-bound after the first APP cleavage by β-secretase. In the homogenates of *Drosophila* brains at 10, 30, and 60 days after eclosion (DAE), both full-length APP and the CTF can be detected (Figure 1A), indicating that the APP was expressed, and that the beta-secretase was functional. Notably, we observed an age-dependent increase of the CTF/FL ratio (Figures 1A,B) and a significant increase of more than double by 60 DAE, indicating continuous APP processing and CTF accumulation in the fly brain over time.

To determine if the CTF accumulation was further processed and able to form amyloid-like plaques in the fly brain, we performed an immunohistochemistry assay using the 6E10 antibody that recognizes amino acids 1–16 of APP and reacts to the abnormally processed isoforms as well as the precursor form. At an early stage (10 DAE), the APP was diffusely expressed in the brain, with a few high-intensity amyloid-like plaques detected (Figure 1C). With aging, at 30 and 60 DAE, we observed a significant increase in plaque number (Figure 1D), consistent with the CTF accumulation over time (Figures 1A,B). Of note, although APP was expressed in all neurons, the plaques were mainly located in the cortex layer of the fly brain, recapitulating the neuroanatomical pattern of APP accumulation in vulnerable regions such as cortical and subcortical layers in *postmortem* human AD brains (Ingelsson et al., 2004). Taken together, our *Drosophila* model of AD demonstrated two key pathological signatures *in vivo*: the biochemical feature of age-dependent APP cleavage and accumulation, and the morphological feature of amyloid plaques deposition in the brain.

### hNmnat1 Reduces Amyloid Plaques Accumulation and APP Cleavage in the Brain

**FIGURE 2:**
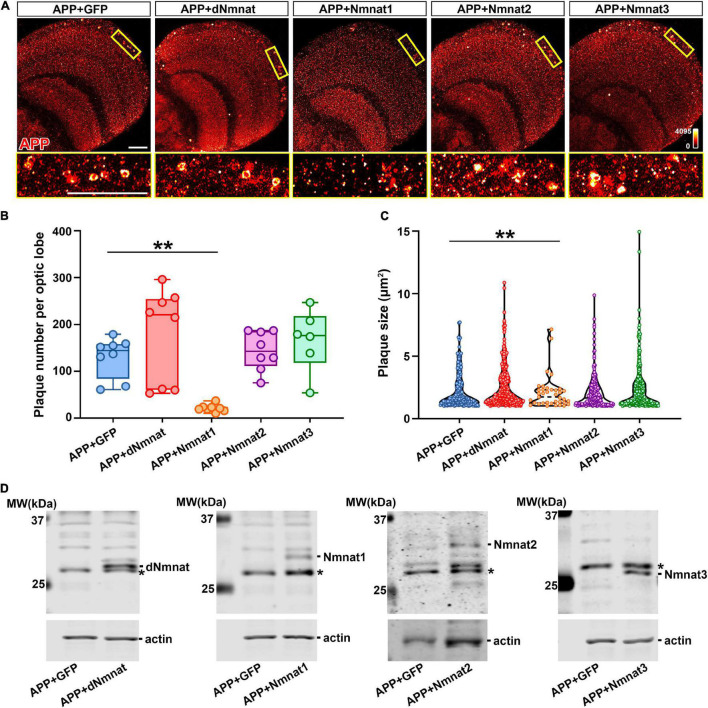
hNmnat1 reduces the accumulation of amyloid plaques in the brain. **(A)** Top row: APP staining (heatmap) of brains of flies expressing APP + GFP, APP + dNmnat, APP + hNmnat1, APP + hNmnat2, or APP + hNmnat3 at 60 DAE. Bottom row: higher magnification images of the boxed areas in the top row. Scale bars = 30 μm. **(B)** Scatter plot showing quantification of the number of amyloid plaques in panel **(A)**. The whiskers represent the minimum and maximum values of the dataset. One-way ANOVA with Bonferroni’s *post hoc* test. *n* = 6–8/group, **P < 0.01. **(C)** Violin plot showing quantification of the size of amyloid plaques in panel **(A)**. One-way ANOVA with Bonferroni’s *post hoc* test. **P < 0.01. **(D)** Western blot analysis showing expression of Nmnat isoforms in fly brains at 60 DAE. Actin is used as an internal control. * Indicates a non-specific band.

We have previously shown that dNmnat and mammalian Nmnat3 protect against neurodegeneration in AD models by chaperoning hyperphosphorylated Tau (pTau) species and ameliorating pathological pTau aggregation (Ma et al., 2020). Moreover, the level of human Nmnat2 has been reported to negatively correlate with the burden of neuritic plaques and neurofibrillary tangles in postmortem human AD brains (Ali et al., 2016). To investigate the effect of different Nmnat isoforms on APP aggregation, we expressed APP together with dNmnat, hNmnat1, hNmnat2, hNmnat3, or mGFP (mitochondrial GFP as control) in the *Drosophila* nervous system and stained for APP at 60 DAE. We found that only the expression of hNmnat1, the nuclear isoform, significantly reduced amyloid plaques deposition in the brain (Figure 2A). Further quantification showed a significant reduction of amyloid plaque number and size in the hNmnat1 overexpression group when compared to those in the mGFP group (Figures 2B,C). We did not observe a significant change of amyloid plaque burden when overexpressing dNmnat, hNmnat2, or hNmnat3. All the overexpressed Nmnat isoforms can be detected in the fly brains (Figure 2D).

**FIGURE 3:**
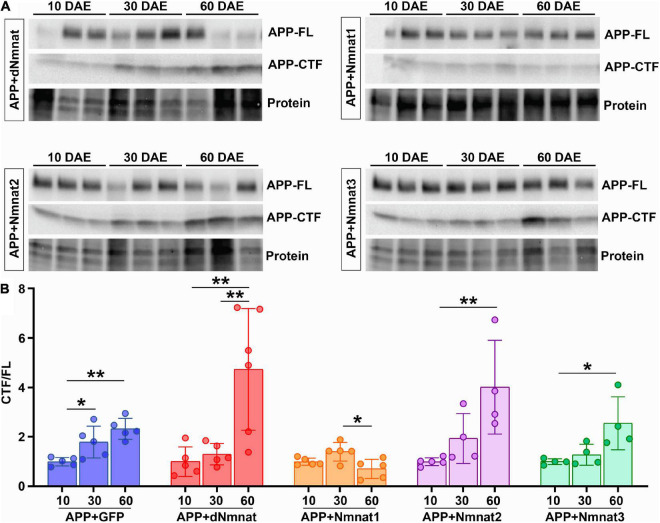
hNmnat1 reduces APP cleavage and production of APP-CTF in the brain. **(A)** Western-blot analysis of full-length amyloid precursor protein (APP-FL) and C-terminal fragment (APP-CTF) of 10, 30, and 60 DAE flies expressing APP + dNmnat, APP + hNmnat1, APP + hNmnat2, or APP + hNmnat3. Stain-Free imaging of a representative portion of the membrane is presented to show the protein loading. **(B)** Quantification of CTF/FL ratio in each group. Data are expressed as mean ± SD. *n* = 4–6/group. One-way ANOVA with Bonferroni’s *post hoc* test. *P < 0.05, **P < 0.01).

Next, we performed biochemical analysis to investigate how different Nmnat isoforms affect APP processing. As we have shown in Figure 1A, neuronal expression of APP led to an age-dependent accumulation of CTF in the brain. When APP was co-expressed with dNmnat, Nmnat2, or Nmnat3, we still observed a significant increase of CTF level with age, while Nmnat1 overexpression remarkably inhibited CTF accumulation (Figures 3A,B). Collectively, our data identified Nmnat1 as a potent inhibitor of amyloid plaque deposition and APP-CTF accumulation.

### Nmnat1 Promotes Amyloid Aggregates Clearance Through Autophagy

**FIGURE 4:**
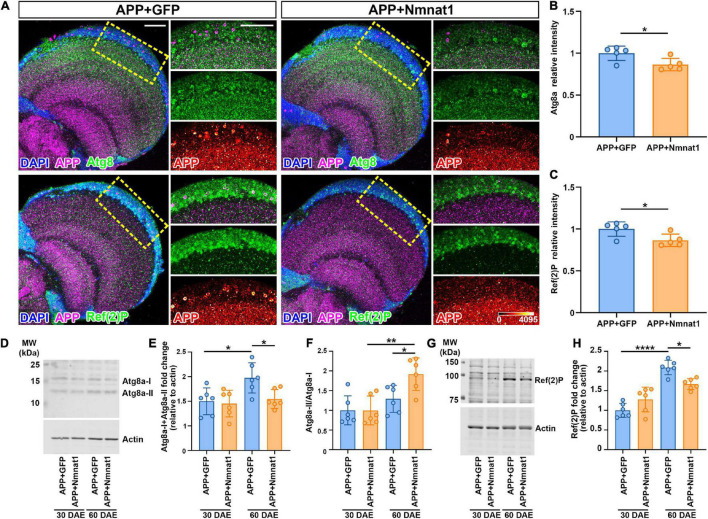
hNmnat1 promotes amyloid aggregates clearance through autophagy. **(A)** Fly brains at 60 DAE were stained with DAPI (blue), APP (magenta), and Atg8 or Ref(2)P (green). Images in the right column are high magnification of the boxed areas (APP is shown in heatmap). Scale bars = 30 μm. **(B,C)** Quantification of relative Atg8a and Ref(2)P intensity shown in panel **(A)**. Data are expressed as mean ± SD. *n* = 5/group. Independent sample *t*-test. *P < 0.05. **(D)** Western blot analysis showing levels of endogenous Atg8a-I and Atg8a-II in fly brains at 30 and 60 DAE. Actin is used as an internal control. **(E,F)** Quantification of total Atg8a level (Atg8a-I + Atg8a-II) **(E)** and the ratio of Atg8a-II to Atg8a-I **(F)**. Data are expressed as mean ± SD. *n* = 6/group. One-way ANOVA with Bonferroni’s *post hoc* test. *P < 0.05, **P < 0.01. **(G)** Western blot analysis showing levels of endogenous Ref(2)P in fly brains at 30 and 60 DAE. Actin is used as an internal control. **(H)** Quantification of Ref(2)P level. Data are expressed as mean ± SD. *n* = 6/group. One-way ANOVA with Bonferroni’s *post hoc* test. *P < 0.05, ****P < 0.0001.

Autophagy has been reported to play a key role in the degradation of Aβ plaques and protects against Aβ-induced neurotoxicity (Hung et al., 2009). To delineate how hNmnat1 inhibits APP-CTF accumulation and amyloid plaques deposition, we applied an immunofluorescence approach to analyze endogenous autophagy genes (Atg)8a, the *Drosophila* homolog of mammalian LC3 that is recruited on autophagosomal membranes (Simonsen et al., 2008). We observed that at 60 DAE, hNmnat1-overexpressing flies exhibited a significant reduction of Atg8a level in the cortex layer of the brain where most APP plaques accumulated (Figures 4A,B). Atg8a in *Drosophila* exists in two forms: an unprocessed cytosolic form (Atg8a-I) and a processed phosphatidylethanolamine-modified form that associates with autophagosomal membranes (Atg8a-II) (Mauvezin et al., 2014). During autophagosome formation, the soluble Atg8a-I is converted into Atg8a-II through covalent binding to phagophore membranes, and Atg8a-II is eventually degraded inside mature autolysosomes (Manzéger et al., 2021). To assess the levels of Atg8a-I and Atg8a-II and the conversion of Atg8a-I to Atg8a-II, we performed biochemical analysis using an anti-GABARAP (γ-aminobutyric acid receptor-associated protein) antibody (Jacomin et al., 2020). GABARAP is a subfamily of Atg8 and the anti-GABARAP antibody has been verified for recognizing *Drosophila* Atg8 (Jipa et al., 2021). Consistent with imaging, we observed an overall reduction of total Atg8a in the fly brains with hNmnat1 overexpression at 60 DAE (Figures 4D,E), as well as a significant increase of Atg8a-II/Atg8a-I ratio, indicating promotion of autophagic flux (Figure 4F; Ratliff et al., 2016). Next, we analyzed Ref(2)P, the *Drosophila* homolog of human p62/SQSTM1, an adaptor protein that tethers ubiquitinated protein, binds to Atg8a, and is degraded in autolysosomes (Bjørkøy et al., 2005; Nezis et al., 2008). In brain imaging, we found a significant decrease of Ref(2)P level in the brain cortex layer at 60 DAE when hNmnat1 was overexpressed (Figure 4C), consistent with western blot analysis (Figures 4G,H). Notably, in the APP + mGFP group, we observed increased levels of total Atg8a and Ref(2)P at 60 DAE when compared to those at 30 DAE (Figures 4D–H), indicating an autophagy defect in older flies as previously reported (Bartlett et al., 2011). Taken together, our data indicated that hNmnat1 reduced the load of amyloid plaques in the brain through the promotion of autophagic clearance.

## Discussion

In this study, we investigated the effect of different Nmnat isoforms on amyloid clearance in a *Drosophila* model of amyloid aggregation. Our model recapitulated the age-dependent processing of APP with CTF accumulation, and deposition of amyloid plaques in the brain cortex. Among different Nmnat isoforms, we identified hNmnat1 as a potent inhibitor for APP aggregation as evidenced by a remarkable reduction of the processed APP-CTF and decreased number and size of amyloid plaques in the brain. Finally, we showed an increased conversion of Atg8a-I to Atg8a-II as well as a reduction of total Atg8a and Ref(2)P in hNmnat1 overexpressing brains, indicating that hNmnat1 reduces amyloid plaque load by promoting autophagy.

Nmnat has been identified as a neuronal maintenance factor (Zhai et al., 2006). Cytoplasmic Nmnat isoforms like dNmnat in *Drosophila* used in this study, hNmnat2, and hNmnat3 are essential for maintaining neuronal integrity in a physiological environment and protecting against neurodegeneration in various neurodegenerative models (Brazill et al., 2017). These Nmnat isoforms can act as chaperones to interact with client proteins, facilitate protein folding and clearance, and thereby maintain proteostasis in neurons (Ali et al., 2016; Zhu et al., 2019; Ma et al., 2020). For example, Nmnat2 forms a complex with HSP90 chaperone to promote refolding of toxic tau (Ali et al., 2016) and Nmnat3 uses its NAD^+^ substrate-binding site to bind and chaperone pTau (Ma et al., 2020).

Aggregates formed from APP processing differ from other protein aggregates in that APP is processed on the plasma membrane and deposited extracellularly (LaFerla et al., 2007). As Nmnat1 is a predominantly nuclear-localized enzyme, it is unlikely for Nmnat1 to directly interact with APP or amyloid plaques. Our findings indicate that hNmnat1 promotes the autophagic clearance of unfolded or misfolded amyloid proteins, likely due to NAD^+^-dependent transcriptional or post-translational regulations. NAD^+^ is a coenzyme involved in hundreds of metabolic redox reactions, ADP-ribosylation, histone deacetylation, and calcium signaling pathways (Brazill et al., 2017). NAD^+^ can be synthesized by a salvage pathway or by a *de novo* pathway and Nmnat plays an essential role in both: it synthesizes NAD^+^ from nicotinamide mononucleotide (NMN) in the salvage pathway or it converts nicotinic acid mononucleotide (NaMN) to nicotinic acid adenine dinucleotide (NaAD) in the *de novo* pathway (Lau et al., 2009). Nmnat1 is essential for the supply of nuclear NAD^+^, a substrate for poly (ADP-ribose) polymerase (PARP)-mediated ADP-ribosylation and sirtuin (SIRT)-mediated deacetylation, both of which are critical regulators of autophagy. For example, PARP1, a ubiquitous nuclear enzyme, catalyzes PARylation of nuclear AMP-activated protein kinase (AMPK), inducing AMPK nuclear-cytosolic export for autophagosome formation and autophagy initiation (Rodríguez-Vargas et al., 2016). Upon DNA damage, PARP1 is required for autophagy induction by increasing the expression of *Bnip-3*, *Cathepsin b and l*, and *Belin-1* (Muñoz-Gámez et al., 2009). We also recently demonstrated that Nmnat1 directly forms a complex with PARP1 and increases local NAD^+^ availability to promote PARylation and regulate cell survival under stress (Liu et al., 2021). In addition, nuclear SIRTs carry out multifaceted functions in autophagy. For example, SIRT1 deacetylates essential components of the autophagy machineries, including Atg5, Atg7, and Atg8, to promote autophagy initiation (Lee et al., 2008). SIRT1 also deacetylates and activates FOXO1 and FOXO3, two essential transcriptional regulators for autophagy induction (Brunet et al., 2004; Zhou et al., 2012). We recently showed that Nmnat1 upregulates SIRT1 and inhibits the activity of p53 (Liu et al., 2021), a negative regulator of autophagy (Tasdemir et al., 2008).

In addition to promoting autophagy, the nuclear NAD^+^ pool regulates the transcription of the major cleaving enzymes involved in APP processing. For example, BACE1 is tightly regulated by peroxisome proliferator-activated receptor-γ coactivator 1 (PGC)-1α, a transcriptional coactivator that enhances BACE1 ubiquitination and proteasomal degradation and ameliorates Aβ production (Gong et al., 2013; Wang et al., 2013). A decreased level of PGC-1α has been reported in the cortex of AD patients, while exogenous expression of PGC-1α can significantly inhibit Aβ plaque formation by suppressing BACE1 transcription (Katsouri et al., 2011). The transcriptional activity of PGC-1α depends on its subcellular distribution and is promoted by SIRT1-dependent nuclear translocation (Anderson et al., 2008). A previous study showed that nicotinamide riboside (NR), an NAD^+^ precursor, upregulates the expression of PGC-1α, enhances BACE1 ubiquitination and proteasomal degradation, and thereby ameliorates Aβ production (Gong et al., 2013).

Taken together, by using a *Drosophila* model of AD, our study demonstrated that overexpressing hNmnat1 inhibits amyloidogenic processing of APP and reduces amyloid plaque accumulation. Recently, high-throughput screens have identified small molecules to boost Nmnat expression in neurons or enhance NAD^+^ production (Ali et al., 2017; Gardell et al., 2019), highlighting the therapeutic potential of targeting Nmnat/NAD^+^ in treating AD and other proteinopathies.

## Materials and Methods

### *Drosophila* Stocks and Genetics

Flies were maintained on cornmeal-molasses-yeast medium at room temperature (RT) with 65% humidity, 12 h light/12 h dark cycles. The following fly strains were obtained from Bloomington *Drosophila* Stock Center: *UAS-APP^695^, UAS-BACE1*; *UAS-GFP*; *elav-GAL4*. The following fly strains were generated in the lab (Zhai et al., 2006, 2008): *UAS-dNmnat*; *UAS-Nmnat1*; *UAS-Nmnat2*; *UAS-Nmnat3*. Only female flies were used in the experiments.

### Protein Extraction and Western Blot Analysis

For analyzing Nmnat expression and autophagy (Figures 2, 4), 10 heads of each genotype were homogenized using radioimmunoprecipitation assay (RIPA) buffer. Extracted samples were mixed with Laemmli sample buffer (2% SDS, 10% glycerol, 62.5 mM Tris–HCl, 0.001% bromophenol blue, and 5% β-mercaptoethanol), and denatured at 95°C for 10 min. Proteins were separated by SDS-PAGE (sodium dodecyl sulfate-polyacrylamide gel electrophoresis) and transferred to a nitrocellulose membrane. After blocking at RT for 1 h, the membrane was incubated with primary antibody at 4°C overnight, followed by secondary antibody incubation for 1 h at RT. The membrane was scanned by an Odyssey Infrared Imaging system (LI-COR Biosciences) and images were analyzed using Image Studio (version 4.0). The following primary antibody dilutions were used: anti-dNmnat [1:1,000, generated in the lab (Zhai et al., 2006, 2008)], anti-Nmnat1 (sc-271557, 1:1,000, Santa Cruz, Dallas, TX, United States), anti-Nmnat2 (ab56980, 1:1,000, Abcam, Cambridge, MA, United States), anti-Nmnat3 (ab71904, 1:1,000, Abcam), anti-Ref(2)P (1:500, Abcam), anti-GABARAP (1:1,000, MBL International Corporation), anti-β-actin (1:10,000, Sigma-Aldrich, St. Louis, MO, United States). The following secondary antibody dilutions were used: IRDye 700DX conjugated anti-Guinea pig (1:10,000, Rockland, PA, United States), DyLight 680 conjugated anti-Rabbit IgG (1:10,000, Rockland), DyLight 800 conjugated anti-Mouse IgG (1:10,000, Rockland).

For analyzing APP processing (Figures 1, 3), heads homogenates were run on a 4–20% gradient Tris–HCl gel (BioRad) and transferred to a PVDF membrane (BioRad). Membranes were blocked in 4% milk in PBST (PBS + 0.1% Tween 20) for 1 h at room temperature. Anti APP C-terminal Fragment C1/6 (Covance SIG-39152) was diluted 1:1,000 in 1% milk in PBST and incubated overnight at 4°C. Goat anti-mouse HRP-conjugated secondary antibody (Cell signaling) was used, and the reaction was developed by chemiluminescence using SuperSignal West reagent (Rockford, IL, United States). Blots were visualized with Chemidoc Imaging System (BioRad). Optical density measurements were taken by software supplied by BioRad. Bands were normalized for the total protein loading (visualized by stain-free technology, in the Chemidoc system, Biorad).

### Brain Dissection, Immunostaining, and Confocal Imaging

Dissection of the *Drosophila* brain was performed in dissection dishes with an elastomer bottom. Dissection was performed in phosphate-buffered saline (PBS). Dissected fly brains were immediately fixed with freshly made 4% formaldehyde for 15 min, washed in PBS containing 0.4% (v/v) Triton X-100 (PBTX) 3 times (10 min each), and incubated with primary antibodies diluted in 0.4% PBTX with 5% normal goat serum at 4°C overnight. Brains were incubated with conjugated secondary antibodies at RT for 1 h, followed by 4′,6-diamidino-2-phenylindole (DAPI, 1:300, Invitrogen, Carlsbad, CA, United States) staining for 15 min. Brains were mounted on glass slides with VECTASHIELD Antifade Mounting Medium (Vector Laboratories Inc., Burlingame, CA, United States). The following primary antibodies were used: anti-APP (6E10, 1:250, BioLegend), anti-Ref(2)P (1:250, Abcam), anti-GABARAP (1:250, MBL International Corporation). The following secondary antibodies were used: Alexa Fluor 555 Goat anti-Mouse IgG (1:250, Thermo Fisher Scientific, MA, United States), Cy5 conjugated anti-Rabbit IgG (1:250, Rockland). Fly brains were imaged using an Olympus IX81 confocal microscope coupled with a 60 × oil immersion objective lens, 1.30 numerical aperture (NA), with a scan speed of 8.0 μs per pixel and spatial resolution of 1,024 by 1,024 pixels (12 bits per pixel). Images were processed using FluoView 10-ASW (Olympus) and Adobe Photoshop CS6 (Adobe Systems). Amyloid plaques were quantified in Fiji/Image J (v1.52).

### Statistical Analysis

Details regarding each statistical test, sample size (n), and *P* value are indicated in the figure legends. *P* < 0.05 was considered statistically significant. All statistical analyses were carried out in GraphPad (v8.0).

## Data Availability Statement

The original contributions presented in the study are included in the article/supplementary material, further inquiries can be directed to the corresponding author/s.

## Author Contributions

MP and RZ conceived the project. YZ, AL, and MP carried out the experiments. YZ, AL, RZ, and MP analyzed the data and wrote the manuscript. All authors contributed to the article and approved the submitted version.

## Conflict of Interest

The authors declare that the research was conducted in the absence of any commercial or financial relationships that could be construed as a potential conflict of interest.

## Publisher’s Note

All claims expressed in this article are solely those of the authors and do not necessarily represent those of their affiliated organizations, or those of the publisher, the editors and the reviewers. Any product that may be evaluated in this article, or claim that may be made by its manufacturer, is not guaranteed or endorsed by the publisher.
